# Influence of the COVID-19 Pandemic on Quality of Life, Mental Health, and Level of Physical Activity in Colombian University Workers: A Longitudinal Study

**DOI:** 10.3390/jcm11144104

**Published:** 2022-07-15

**Authors:** Patricia Alexandra García-Garro, Agustín Aibar-Almazán, Yulieth Rivas-Campo, Gloria Cecilia Vega-Ávila, Diego Fernando Afanador-Restrepo, Fidel Hita-Contreras

**Affiliations:** 1 GIP Pedagogy Research Group, Faculty of Distance and Virtual Education, Antonio José Camacho University Institution, Santiago de Cali 760016, Colombia; palexandragarcia@admon.uniajc.edu.co (P.A.G.-G.); julieth519.jr@gmail.com (Y.R.-C.); gcvega@profesores.uniajc.edu.co (G.C.V.-Á.); dafanador@profesores.uniajc.edu.co (D.F.A.-R.); 2 Department of Health Sciences, Faculty of Health Sciences, University of Jaén, 23071 Jaén, Spain; fhita@ujaen.es

**Keywords:** physical activity, sleep quality, depressive symptoms, quality of life, lockdown, COVID-19

## Abstract

The COVID-19 pandemic has had a strong influence on people’s quality of life, and the different restrictive measures during the phases of the pandemic have had consequences for physical and mental health. This study determined the changes in health-related quality of life (HRQoL), sleep quality, depression symptoms (DS), and physical activity (PA) level of middle-aged Colombian university personnel during the COVID-19 pandemic. The analysis was performed following a longitudinal design on a sample of 354 people at four points before and during the pandemic. The International Physical Activity Questionnaire (IPAQ), the SF-12v2, the Pittsburgh Sleep Quality Index (PSQI), and the Zung Self-Rating Depression Scale (ZSDS) were included in an online survey. The measurements showed a decrease in quality of life with respect to PCS from 91.66 ± 10.78 to 83.64 ± 17.22 (*p* = 0.000) and MCS from 87.57 ± 12.23 to 80.90 ± 17.31, while poor sleep quality increased from 25.99% to 47.46% (*p* = 0.000), with DS reaching the peak during mandatory confinement (14.69%). Regarding the level of physical activity, the period of mandatory confinement inverted the proportion, highlighting active people as a minority (32.2%). In the multivariate analysis, we adjusted for age, sex, BMI, and socioeconomic level, discarding confounding effects and their interactions with the results obtained. In conclusion, this study showed that the pandemic has had a negative impact on sleep quality, HRQoL, DS, and PA level.

## 1. Introduction

COVID-19, which in 2022 continues to constitute a public health emergency of international concern [1], was declared a pandemic on 11 March 2020 by the World Health Organization (WHO) [2]. This pandemic drastically altered the daily lives of people around the world [3,4] and brought changes to the labor dynamics; according to the International Labor Organization (ILO), by the second quarter of 2020, around 23 million people in Latin America and the Caribbean switched to teleworking [5]. Likewise, during the mandatory preventive isolation period, the personnel working in Colombian universities switched from on-site work to remote work, working at home, teleworking, or similar modalities [6], and then to a hybrid modality between remote and on-site work, called “alternating education”, which was subject to strict biosafety protocols and capacity control [7], until the beginning of 2022, when the total return to on-site work was decreed with full capacity [8].

These practices have generated harmful situations for workers, such as presenteeism, a perception of increased workload, and even physical and mental health problems in Colombian professionals [9]. In addition, Staniec et al. [10] found that, during the first 3 months of the COVID-19 pandemic, the emotional experiences of working from home were similar between people with pre-pandemic remote work experience and people who had to migrate to the modality.

Moreover, in relation to health and wellbeing, it was reported that the pandemic initially intensified poor sleep quality [11], depression [12], and physical inactivity [13], a situation of great concern because these are three major global public health problems [14,15,16] impacting people’s quality of life [17].

As a result, during confinement, sleep habits were altered [18], leading to a poor quality of sleep [11]. A study conducted in Italy with students and university administrative staff found that the prevalence of insomnia in workers increased during mandatory confinement from 24% to 40%, and difficulties in initiating sleep increased from 15% to 42% [19]. Likewise, a study conducted with Chilean teachers showed that quality of life decreased significantly during the pandemic compared to the pre-pandemic measurement [20], while a study conducted in Indonesia, in which 76% of those evaluated worked from home during the pandemic, found that the lowest level of quality of life was in the social domain [21].

In the first waves of the pandemic, during mandatory confinement, people presented a deterioration in their mental health [22,23]; according to the WHO, the pandemic triggered a 25% increase in the prevalence of depression worldwide [24]. Furthermore, according to Keisari et al. [25], several depressive symptoms persisted despite the removal of social restrictions.

On the other hand, when comparing physical activity (PA) levels prior to the pandemic and during the first wave of confinement, it was found that people spent more time on sedentary behaviors (~75 min/day) and on moderate intensity activities (~10 min/day) [26]. Similarly, changes in vigorous and moderate PA levels were reported during and after confinement, compared to pre-confinement levels; such changes were dependent on the PA level of pre-COVID-19 individuals [27].

In relation to the evolution of changes in mental and physical health during the COVID-19 pandemic, most studies compared data pre pandemic/lockdown [26], at different times of confinement [28], and lockdown/post lockdown [27,29]. Additionally, to our knowledge, most studies addressing these issues have been cross-sectional [30], focused mainly on health care workers [31], with few longitudinal studies addressing these issues [32], and no study analyzing together the changes in different variables of mental and physical health in the middle-aged Colombian population pre-pandemic and at different times of the pandemic. COVID-19 represents a new labor challenge worldwide; in addition to the context in which university personnel are found, the work overload that has been worsened by teleworking and other factors have generated deterioration in the quality of life of this population [20].

Therefore, this study aimed to determine changes in the quality of life, sleep quality, depression, and physical activity level in middle-aged Colombian university personnel during the COVID-19 pandemic. In this way, the levels of physical activity, depressive symptoms, sleep quality and quality of life of university personnel are expected to worsen during mandatory confinement and to improve gradually in the time following the COVID-19 pandemic.

## 2. Materials and Methods

### 2.1. Study Design and Participants

A longitudinal study was conducted with university personnel from higher-education institutions in Cali, Colombia, with a total of 354 participants. The subjects were people teaching or administration and services staff of the universities between 40 and 64 years old. The invitation to participate in this study was made through the lists of e-mails of the university workers, obtained from the area of occupational safety and health. This invitation was made through an email in which the participants received detailed information about the study both in writing and by video.

Data were collected through an online questionnaire designed on the Google Forms platform due to the COVID-19 pandemic at three different times. The first phase was during the mandatory preventive isolation (T1) between 26 May and 23 June 2020, which was characterized by the total limitation of free movement of people [33], in addition to the transition from face-to-face to remote work modalities, work at home, telework, or similar [6]. In this first data collection, participants were asked to complete the questionnaires twice, one in which they answered the questions relating them to the current period and another about the period before the pandemic (T0). The second data collection was performed during selective isolation and responsible individual distancing (T2) between 26 February and 31 March 2021. In this phase, voluntary self-isolation, limitation of public or private events, and alternating education, with strict control of capacity and crowding of people, predominated. Lastly, the third data collection took place during selective isolation with responsible individual distancing and safe economic reactivation (T3), from 2 December 2021 to 8 February 2022. This phase implied the progressive return to normality by gradually eliminating capacity and mobility restrictions and physical distancing and de-escalating the compulsory use of masks, as well as reactivating the economic, social, and state activities [34], thus returning to the “new normal” under the corresponding biosecurity protocols.

Before enrolment in the study, all participants received a booklet with detailed information about the study and gave their written consent. This study was approved by the Research Ethics Committee of the Antonio José Camacho University Institution (FEV-001-21-01).

### 2.2. Sample Size Calculation

The calculation of the sample size and power [35] was performed using the EPIDAT 4.1 platform (Dirección Xeral de Innovación e Xestión da Saúde Pública de la Consellería de Sanidade with the support of the Pan American Health Organization, Coruña, Spain), with a 95% confidence interval, precision of 5%, and a population size of 973. Accordingly, the minimum number of cases required for a statistical power of 80% was 171 persons; taking into account the potential losses during follow-up (25% loss in each measurement), it was adjusted to 300. Finally, the randomization of the sample was obtained from a consolidated list of the institutions in which each participant was assigned a number; subsequently, using again the EPIDAT 4.1 platform, a simple randomization was performed, which resulted in a total of 378 people that were invited to participate in the study.

### 2.3. Outcomes

All of the variables conducted in this study were measured using an online form due to the current global situation.

#### 2.3.1. Sociodemographic and Anthropometric Data

Multiple questions were asked about the demographic data of the participants, including age, sex (male or female), marital status (single/free union, married, separated/widowed), socioeconomic strata, which are classified by Colombian law 142 of 1994 by numbers (1–6 according to the quality of facilities and services available), residence (urban or rural: <150 inhabitants per km^2^ [36]), smoking habit (no or yes), alcohol consumption (no or yes; when answering yes, the participant should specify the frequency of consumption, being frequently when it was weekly or occasionally if it was bi-weekly), diagnosis of COVID-19 (no or yes) and living with people at risk of COVID-19 (no or yes). People at risk of COVID-19 were defined as housemates or family members at high risk of contracting COVID-19 or at risk of developing severe or fatal COVID-19. Weight and height were obtained from the latest update of the databases of the occupational health and safety areas of the universities that took part in the study. These updates are made with the control occupational medical examinations. Finally, with these last two data, the body mass index (BMI) was obtained.

#### 2.3.2. Quality of Life Related to Health

Health-related quality of life (HRQoL) was assessed using the SF-12v2 questionnaire, validated in the Colombian population (Cronbach’s alpha = 0.7) [37]. This questionnaire, composed of 12 items, is divided into eight domains or scales: (i) physical role, (ii) physical functioning, (iii) general health, (iv) bodily pain, (v) social functioning, (vi) vitality, (vii) mental health, and (viii) emotional role. In addition, two general scores were calculated: physical health component summary (PCS) and mental health component summary (MCS). For each domain, the score was graded from 0 to 100, where higher scores correlated with a better HRQoL. For the dichotomization of this variable, the reference value for the Colombian population was taken as the cut-off point, classifying those with scores <50 as below average and ≥50 as above average [37].

#### 2.3.3. Sleep Quality

The Pittsburgh Sleep Quality Index (PSQI) was used to assess the participants’ sleep quality [38]. This instrument was previously validated in a Colombian population (Cronbach’s alpha = 0.78) [39]. The PSQI is composed of 24 questions and measures seven different domains: (i) sleep latency, (ii) subjective sleep quality, (iii) daytime dysfunctions, (iv) sleep duration, (v) sleep disturbances, (vi) habitual sleep efficiency, and (vii) use of sleep medications, generating a global score. Each domain can be scored between 0 and 3 points, resulting in a global score ranging from 0 to 21, where higher scores are related to worse sleep quality [38,39,40]. Additionally, this variable was recategorized into two groups: with low sleep quality (LSQ) and without LSQ, taking a global score of 5 as the cutoff point, as applied in other studies [41,42].

#### 2.3.4. Depression

Depression was assessed using the Zung Self-Rating Depression Scale (ZSDS) [43], which has been validated for the Colombian population (Cronbach’s alpha = 0.85) [44]. This instrument consists of 20 questions split into 10 positive and 10 negative questions related to the frequency of depressive symptoms (DS) during the last 2 weeks. Each question receives a score between 1 and 4 (a little of the time = 1; some of the time = 2; good part of the time = 3; most of the time = 4), which means that the total score can range between 20 and 80 points, where higher scores are related to the presence of DS. For dichotomization, a global score of 55 points was taken as the cutoff point, resulting in two categories: with DS (>55) and without DS (≤55) [45].

#### 2.3.5. Level of Physical Activity

The International Physical Activity Questionnaire (IPAQ) validated in the Colombian population (Cronbach’s alpha = 0.64) was used to measure the level of physical activity (PA) [46]. This questionnaire measures the time that an individual has spent engaging in PA during the last 7 days, classifying it by levels on the basis of three qualities: the frequency, intensity, and duration of the PA practiced. These three qualities result in a measurement in Mets, which allows the subject to be placed in three different categories: (i) high, 1500 Mets·min/week of vigorous PA or 3000 Mets·min/week of moderate physical activity, three or more times per week; (ii) moderate, 600 Mets·min/week of moderate PA or vigorous PA for at least 25 min, three or more times per week; (iii) low, when the participant does not perform PA or the PA performed is not enough to be classified in the other two categories [47]. Additionally, the variable was recategorized as active (high and moderate classification) or insufficiently active (low classification) [48].

### 2.4. Data Analysis

The STATA 14 program was used for all analyses. An exploratory analysis was performed to determine the normality of the distribution of the variables using the Kolmogorov–Smirnov test. The sociodemographic characteristics and quantitative variables such as MCS and PCS for the SF-12 and the PSQI domains and total score were described by means and standard deviations. Likewise, the outcomes from SF-12, IPAQ, ZSDS, and PSQI were dichotomized and presented as frequencies and percentages for each of the four measurement periods. The dichotomized variables were analyzed with contingency tables, performing a test of homogeneity (equal odds) through the χ^2^ test; Cramer’s V was calculated (≤0.2 effect size is weak; 0.2–0.6 result is moderate; and >0.6 effect size is strong). Additionally, the reliability of the data obtained in the SF-12, the PSQI and the ZSDS was measured based on Cronbach’s alpha, considering values above 0.7 as acceptable [49,50]. The association of these variables with the time of measurement was evaluated using a logistic regression model and adjusted for sex and age. The MCS and PCS from SF-12 and the PSQI total score were also analyzed quantitatively; differences between baseline and follow-up for sleep quality and quality of life were examined using the measures repeated ANOVA test, and Bonferroni adjustment was applied. The effect size was measured with Cohen’s *d* (0.2 small, 0.5 medium and 0.8 large) The confusion or interaction was evaluated through the analysis of covariance ANCOVA, which allowed us to determine whether there were significant differences in sleep quality and quality of life at each measurement stage adjusted for age, sex, socioeconomic stratum, marital status, alcohol consumption, being a smoker, BMI, being diagnosed, with COVID-19 and living with people at risk of COVID-19.

Finally, a multivariable analysis of variance was performed through a MANOVA model to evaluate the effect of the measurements (T0, T1, T2 and T3) on sleep quality and quality of life. Significance was assessed with Wilks’ lambda. This model was post-estimated with an eigenvalue analysis and multivariate regression to assess whether the matrix values and eigenvalues were real and supported the impact of the time of measurement of these outcome variables. The acceptable threshold of statistical significance was specified as 0.05.

## 3. Results

**Figure 1:**
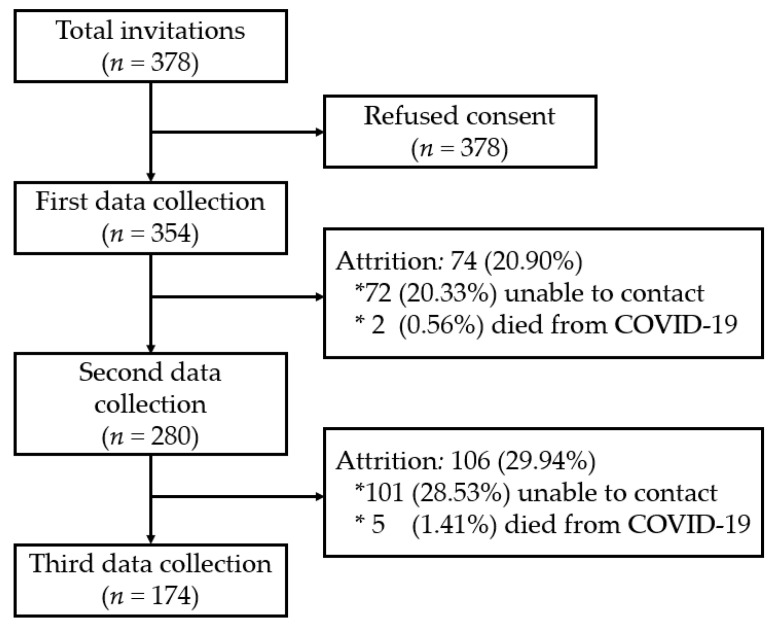
Flow chart of the study participants.

A total of 378 subjects were approached to participate in the study. Twenty-four of these subjects declined informed consent, while 354 decided to participate voluntarily during the first data collection period (T0 and T1). During the second data collection period (T2), 280 subjects were surveyed, losing 74 participants, of which 72 were impossible to contact, while two died due to COVID-19. During the third data collection period (T3), 106 participants dropped out of the study, of which 101 were impossible to contact, and five died from COVID-19 or other causes, leaving a total of 174 people surveyed at this point (Figure 1).

Table 1 presents the descriptive data of the participants. A total of 354 subjects were surveyed, with a predominance of male sex (59.04%) and an average age of 43.39 (SD = 10.21). BMI was categorized on the basis of WHO reference parameters, showing a predominance of overweight (49.44%) and normal-weight (37.29%) subjects. In addition, 55.37% of the participants were single or in free union, while 34.46% were married, and only 10.16% were divorced or widowed. Regarding residence, most of the population lived in the urban area (86.44%) in a middle socioeconomic stratum comprising strata 3 (31.92%) and 4 (32.49%). It was also evident that most of the sample had children (66.10%), were nonsmokers (95.76%), and consumed alcohol occasionally (63.56%); only 33.05% lived with people at high risk of COVID-19.

**Figure A1:**
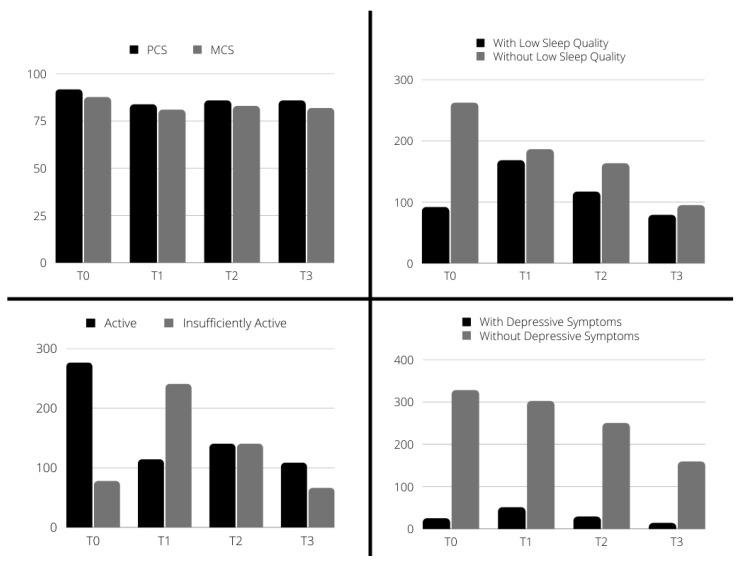
Changes in PCS, MCS, sleep quality, BP and depressive symptoms at different data points. T0: period before the pandemic; T1: mandatory preventive isolation; T2: selective isolation and responsible individual distancing; T3: selective isolation with responsible individual distancing and safe economic reactivation; PCS: physical health component summary; MCS: mental health component summary.

Figure A1 shows the changes in the outcome variables at the different times of data collection (Appendix A). Table A1 shows the Cronbach’s alpha of the instruments used in each data collection (Appendix B).

Table 2 shows the results of the variables for each measurement stage (T0, T1, T2, and T3) and the statistical evaluation of their differences by repeated measures ANOVA test. The quantitative analysis carried out using the SF-12 to evaluate HRQoL allowed us to evidence changes regarding the PCS with a large effect size (Cohen’s *d* = 0.95), which presented a mean of 91.66 ± 10.78 during T0, indicating a high score in physical health, but decreased to 83.64 ± 17.22 during T1; this value rose slightly at T2 (85.71 ± 14.16) and at T3, but failed to reestablish the initial values (85.78 ± 14.97) (F = 19.85, *p* = 0.000).

On the other hand, the MCS at T0 showed a high score in mental health (87.57 ± 12.23), while the mean decreased slightly at T1 (80.90 ± 17.31), indicating a decline in health-related quality of life in the mental domain. At T2, the MCS went up (87.87 ± 16.46), while, at T3, it went down again (81.75 ± 19.46). This fluctuation represented significant differences during follow-up with a median effect size (d = 0.69). Independent analysis of each domain showed significant differences between measurements, except for social functioning (F = 11.47, *p* = 0.119).

The quantitative results for sleep quality assessed with PSQI indicated that significant differences existed between measurements for six out of seven domains: sleep quality (F= 5.66, *p* = 0.000), sleep duration (F = 5.89, *p* = 0.0005), sleep efficiency (F = 5.45, *p* = 0.001), sleep disturbances (F = 4.24, *p* = 0.005), daytime dysfunction (F = 4.47 *p* = 0.0040) with a median effect size; and sleep latency (F = 12.86, *p* = 0.000) with a large effect size. The total score showed a higher mean at T1 (4.54 ± 3.80) and T3 (4.55 ± 3.78), translating into greater sleep disturbances during these times (F = 10.50 *p* = 0.000 and a large effect size =0.86). The Bonferroni adjustment allowed us to identify that differences were evident with respect to baseline measurements: T0 vs. T1 (*p* = 0.000), T0 vs. T2 (*p* = 0.001) and T0 vs. T3 (*p* = 0.000).

Similarly, the categorical analysis of the sleep quality assessed with the PSQI showed differences between each measurement (*p* = 0.000) with a moderate association (Cramer’s V = 0.201). At T0, LSQ was present in 25.99% of the study population, while, at T1, participants began to report an increased level of sleep problems (47.46%), showing the highest level evidenced during this study. During T2, it started to decrease slightly (41.79%); however, at T3, sleep problems seemed to persist and increase (45.40%), without reaching the levels found at T1 (Table 3).

Regarding the results related to depressive symptoms assessed with the Zung scale, in all measurements, higher proportions of people without depression were identified, but the percentages varied. At T0, low levels of depression were identified (7.34%); at T1, the peak DS of the entire follow-up was reached (14.69%), while at T2, it began to decrease but remained above the initial measurement (10.71%). Lastly, at T3, it continued to decrease, but there were still important levels of depression (8.62%); these data showed statistically significant differences (*p* = 0.012), although the size of the effect indicated that the association was weak (Cramer´s V = 0.097). This situation was repeated with the dichotomous classification of sf12, which despite significant differences (*p* = 0.02) showed a small effect size (Cramer’s V = 0.11) in all measurements, with the highest proportion found in the classification below the mean.

The level of PA evaluated through the IPAQ showed that, at T0, the majority (77.97%) were classified as active; however, this percentage dropped drastically and became a minority at T1, reaching a critical level (32.2%), before leveling off (50%) during T2 and finally regaining the majority at T3 (62.07%), albeit remaining far from the initial level. This shows that the level of physical activity presented the highest levels in the pre-pandemic period. These differences between the measurement periods were statistically significant (*p* = 0.000) with a moderate effect size (Cramer’s V = 0.366) (Table 3).

The existence of significant differences between each group of sociodemographic variables that could cause confusion or interaction was evaluated through the analysis of covariance. The objective was to determine whether there were significant differences in sleep quality and quality of life at each measurement stage adjusted for age, sex, socioeconomic stratum, marital status, alcohol consumption, being a smoker, BMI, being diagnosed with COVID-19, and living with people at risk of COVID-19 (Table 4).

Significant differences were discarded when categorized by each of the sociodemographic variables (*p* > 0.05). This indicates that the differences between the measurements (T0, T1, T2 and T3) for sleep quality and quality of life were maintained independently of the sociodemographic characteristics of the population; therefore, their confounding effects and interactions with the results obtained were ruled out.

The MANOVA model allowed us to evaluate the effect of the measurements (T0, T1, T2 and T3) on all of the dependent variables showing their significance (Wilks’ lambda *F* = 10.73, *p* = 0.000). The eigenvalues were greater than one (13.17); therefore, we could conclude that the time of measurement did have an impact on the level of quality of life and quality of sleep. The post-MANOVA analysis was carried out with the total values of each outcome (total average physical value and mental value of the quality of life and the total value of the PSQI score) from a multivariate regression, which was able to verify that the matrix values and the eigenvalues were real and supported the impact of the time of measurement of these variables’ results (*p* > 0.000 in all the variables), identifying that in the confinement period (T1) the average of the physical component of quality of life decreased 8 units, while the average of the mental component of quality of life decreased 6 units and sleep disturbance quality increased (1.29 units more in the overall PSQI score) (Table 5).

For each dichotomous outcome (PSQI categorical, IPAQ and ZSDS) a logistic regression model was generated (Table 6). Through this model, the associations with low sleep quality, depression, and being physically active with each measurement stage, adjusted for sociodemographic variables, were studied. This information is summarized in Table 5. The results indicate that regardless of sex, age, and socioeconomic level, being in T0 was a protective factor for low sleep quality (OR 0.35, 95% CI 0.27–0.44, *p* = 0.000). On the other hand, T1 favored low sleep quality by a factor of 2.57 times (OR 2.57, 95 CI, 1.87–3.52), with this association receding slightly in T2 (OR 2.04, 95% CI 1.46–2.86 *p* = 0.000), but again being exacerbated in T3 (OR 2.36, 95% CI 1.6–3.4, *p* = 0.000).

In T1, a strong and significant association was found with the presence of DS (OR 2.17, 95% CI 1.32–3.56, *p* = 0.002), although this estimate was reduced to 51% in T2 and to 19% in T3; these last associations did not present statistical significance (OR 1.51, 95% CI 0.87–2.62, *p* = 0.140; OR 1.9, 95% CI 0.61–2.3, *p* = 0.607, respectively). It should be noted that the chance of developing depression in T0 was 0.07 times, or 93% lower than in the other periods (OR 0.07, 95% CI 0.05–0.11, *p* = 0.000).

The analysis of the level of PA in the studied population indicated that the chance of being active in T0 was 3.5 times higher than in the other periods (OR 3.5, CI 95% 2.75–4.54, *p* = 0.000). This relationship was completely altered in later measurements; the chance was not only reduced but also changed its effect, showing that, in T1, the chance of being active was 0.13 times or 87% lower (OR 0.13, 95% CI 0.09–0.18, *p* = 0.000); in T2, it was 72% lower (OR 0.28, 95% CI 0.2–0.39, *p* = 0.000); and in T3, it was 54% lower (OR 0.46, 95% CI 0.31–0.68, *p* = 0.000).

Lastly, the dichotomized analysis of SF12 did not show significant changes in the T1 and T2 measurements, but did identify a greater chance of having a score above the average in the last period (T3), which coincided with the end of confinement.

## 4. Discussion

The aim of this study was to determine changes in HRQoL, sleep quality, DS, and PA level in middle-aged Colombian university personnel during the COVID-19 pandemic. This study confirmed that mandatory isolation decreased HRQoL and PA level, in addition to reducing sleep quality and increasing DS, with respect to pre-pandemic levels. In addition, differences were detected in the evolution of these variables, and it was found that, in the last screening performed, they had not been reestablished with respect to their initial values. Taken together, these findings clarify the impact of the COVID-19 pandemic on the mental and physical health of the Colombian population and may help in the design of health and wellbeing promotion programs that aim to restore the health of the general population.

In relation to variations in the quality of sleep, this study showed significant changes in which sleep problems were maintained throughout the COVID-19 pandemic. This coincides with other studies suggesting that sleep quality worsened during the COVID-19-generated quarantine [51,52]. Similarly, a study conducted in workers suggested that those who switched to remote work had a 0.7 point (2.8%) increase in worsening sleep quality [53]. At this stage of the pandemic, people’s sleep habits may have changed due to different factors such as reduced exposure to sunlight, reduced physical activity, and psychological distress [52,54].

Furthermore, this research presented an alarming prevalence of people with LSQ during compulsory isolation that persisted beyond. These findings are consistent with those reported by Gorgoni et al. [55], who suggested that strict restrictive measures were not the main cause of sleep problems during the pandemic, and that home confinement induced long-lasting effects on sleep that were observable after its termination; however, further studies are needed to elucidate the causal effects of these factors.

On the other hand, HRQoL showed that scores decreased significantly during mandatory confinement and gradually began to reestablish, but did not reach baseline values.

One cause of this outcome may be the influence of telework on the health of workers, as it has been reported as a factor impacting psychosocial and physical health and burnout due to stress and job exhaustion among employees [56,57,58]. Despite the fact that the outbreak of COVID-19 has had an immediate impact on the physical and mental health of the population [53,59], it seems that there is an adaptation phenomenon, which had already been suggested in previous studies [60,61]. Social function was the only variable that did not show significant differences between the measures of health-related quality of life, which could be explained by the fact that, during the pandemic, many people spent their time at home chatting with friends or on social networks [62].

In addition, we found that the period of compulsory isolation was significantly associated with the presence of depressive symptomatology, which is consistent with the findings of multiple investigations in different populations [63,64]. In the general population, during the first wave of COVID-19, deterioration in mental health was associated with several factors, including fear of infection and of infecting family members [65], as well as preoccupation associated with media information [66,67]. Moreover, for teleworkers, workplace loneliness, low levels of control over working hours [68], as well as changes in working hours, mental work overload, excessive noise and poor internet connection, generated difficulties in developing this modality of work and contributed to a deterioration in mental health [66] and an inability to harmonize family and work life [69]. Moreover, with the progressive removal of social restrictions, this association was gradually reduced to the point that the prevalence of DS before compulsory isolation and during the economic revival reached values similar to the pre-pandemic stage. Similarly, in the Japanese population (30–49 years) [70], a decrease in the occurrence of depression between confinements corresponding to the first and second waves of COVID-19 was reported, from 20.5% to 17.2%, respectively. Otherwise, countries such as the Netherlands reported that the pandemic did not negatively affect the prevalence of depressive symptomatology or the normal recovery from DS among the general population [71]. However, in the Spanish population [72], the negative effects of the pandemic on the increase in DS were exacerbated 1 year after the onset of the pandemic. Moreover, in the Israeli population (*n* = 293, aged 40–85 years) [25], following the removal of social restrictions, people had difficulty adjusting to the return to routine (44% with moderate to high levels of difficulty), and higher levels of adjustment difficulty were associated with higher levels of DS (β = 0.15, *t* = 2.34, *p* < 0.05). Furthermore, middle-aged adults presented higher levels of depressive symptoms and adjustment difficulty (48.7% and 24.7%, respectively) compared to older adults.

In relation to changes in work dynamics during the pandemic, it was reported that front-line healthcare workers showed several signs of mental health deterioration and increased occurrence of depression between confinements corresponding to the first and second waves of COVID-19 [73]. Similarly, employees from other areas, such as those in our research, showed a similar trend; Chilean teachers [20] exhibited a reduced SF-36 MCS score, when comparing values before the pandemic and during the pandemic (42.074 ± 9.68 and 34.959 ± 10.30, respectively, *p* < 0.001). Additionally, assessments of US workers (*n* = 115, aged 45.4 ± 12.3 years) [53] showed that, during home confinement, there was an increase of 1.5 ± 3.9 (*p* < 0.001) points for depression compared to pre-pandemic values.

Moreover, cross-sectional studies on the mental health of workers showed that, during the pandemic, associated factors and predictors for the presence of DS were being a woman [74,75,76,77], preexistence of mental health problems, being younger than 45 years, working at home part-time [74], and perceived job instability [76]. In relation to the previously described factors that influence the presence of DS, it should be noted that the multivariate MANOVA analysis and logistic regression carried out in this study allowed us to rule out sociodemographic variables, including sex, as confounding and interacting with the results obtained. It is possible that our results can be explained to some extent by adaptation and resilience processes in the population [78,79].

During the pandemic, factors such as having hobbies, performing household chores, and the level of organization in the family were associated with a good state of resilience [78]. Furthermore, social capital, understood as the support received, as well as trust in people and institutions, represented a predictor of good resilience state, providing protection against the negative mental health consequences of COVID-19 [79]. Moreover, in this regard, it should be considered that our study population retained employment during the pandemic, although both unemployment and job insecurity have been reported as factors associated with the development of different mental health afflictions during COVID-19, including depression [80,81,82]. Only a small proportion of those included in our investigation (1.41%) developed COVID-19 infection during the pandemic, which is relevant, as 10–65% of COVID-19 survivors (mild/moderate) may develop post-COVID-19 syndrome [83], which has been associated with the development of DS (11–28%) and clinically significant/severe DS (3–12%) at 12 weeks post infection [84].

In the population studied, it was found that the opportunity to be active in the pre-pandemic period was 3.5 times higher than in the other measured periods and that the period of compulsory isolation significantly affected the level of PA. Therefore, a higher proportion of people performed low- and moderate-intensity activities, which is consistent with the report of Cheval et al. [26], who found that French and Swiss people spent more time in sedentary behaviors (~75 min/day) and moderate-intensity activities (~10 min/day) during the first wave of confinement. Likewise, PA in Japanese workers was negatively affected by the COVID-19 outbreak; the time (h/day) spent on vigorous PA before and during confinement averaged (SD) 0.21 (1.03) and 0.19 (0.84), while time spent on moderate PA before and during confinement was 0.43 (1.32) and 0.39 (1.17), respectively. This suggests that teleworking promoted sedentary lifestyles in this population [85]. However, according to our research, this drastic effect on PA levels presented a gradual improvement without reaching pre-pandemic levels; the opportunity to be active was 54% lower during economic reactivation, in contrast to the report of Hargreaves et al. [27], who found that changes in PA levels depended on the level of pre-pandemic PA. In individuals who were very active before confinement, vigorous/moderate PA decreased during and after confinement, whereas in individuals moderately active before confinement, vigorous/moderate PA increased during and after confinement; in both cases, the comparison was made with pre-confinement values.

This study had some limitations. First, the survey responses were self-reported, which could have generated a recall bias; however, applying the same instrument on different occasions strengthened the results. Second, some variables that could partially explain our findings were not taken into account, such as anxiety or work stress. Third, the small sample size could be considered a limitation, and the data were only collected in Cali, so these results are not generalizable to the entire population and should be interpreted with caution. Finally, since the data were collected only in Colombia, it is not possible to make comparisons of the variables between countries, which would add value to this study.

## 5. Conclusions

This study confirmed that the mandatory preventive isolation generated during the COVID-19 pandemic decreased health-related quality of life, PA level and sleep quality, and increased depressive symptomatology, with respect to pre-pandemic levels in Colombian university personnel. In addition, significant differences were found in the evolution of these variables, without restoring them to their initial values.

Taking into account that the pandemic still continues, the present results could be used as a basis for the generation of integral intervention programs in order to diminish the negative impact of COVID-19 on the well-being and quality of life of Colombian university personnel, as well as to support decision making regarding public policies particularly relevant to the physical and mental health of the working population in times of pandemic.

## Figures and Tables

**Table 1:** Descriptive data of the participants (*n* = 354).

Overall Participants (*n* = 354)			
Age, mean (SD)			43.39 (10.21)
Sex, *n* (%)	Male		209 (59.04)
	Female		145 (40.96)
* BMI, *n* (%)	Underweight		3 (0.85)
	Normal range		132 (37.29)
	Overweight		175 (49.44)
	Pre-obese		30 (8.47)
	Obese class 1		7 (1.98)
	Obese class 2		7 (1.98)
	Obese class 3		0 (0.00)
Marital status, *n* (%)	Single/free union		196 (55.37)
	Married		122 (34.46)
	Separated/widowed		36 (10.16)
Residence, *n* (%)	Rural area		48 (13.56)
	Urban area		306 (86.44)
** Socioeconomic stratum, *n* (%)	1		20 (5.65)
	2		54 (15.25)
	3		113 (31.92)
	4		115 (32.49)
	5		47 (13.28)
	6		5 (1.41)
Children, *n* (%)	No		120 (33.90)
	Yes		234 (66.10)
Smoker, *n* (%)	No		339 (95.76)
	Yes		15 (4.24)
Alcohol consumption, *n* (%)	No		129 (36.44)
	Yes		225 (63.56)
		Occasionally	211 (59.60)
		Frequently	14 (3.96)
Diagnosis with COVID-19, *n* (%)	No		349 (98.59)
	Yes		5 (1.41)
*** Living with people at risk of COVID-19, *n* (%)	No		237 (66.95)
	Yes		117 (33.05)

BMI (kg/m^2^): body mass index; IPAQ: International Physical Activity Questionnaire. * According to the WHO classification. ** According to Law 142 of 1994 that establishes the Regime of Domiciliary Public Services in Colombia. SD: standard deviation. *** Housemates or family members with high risk of contracting COVID-19 or are at risk of developing severe or fatal COVID-19.

**Table 2:** ANOVA repeated measures. Differences between measurement times for quantitative variables: HRQoL and sleep quality.

		T0 (*n* = 354)	T1 (*n* = 354)	T2 (*n* = 280)	T3 (*n* = 174)	*df*	*F*	*p*	Effect Size *(d)*
SF-12v2, mean (SD) HRQoL	Physical functioning	94.84 (13.95)	89.90 (21.17)	89.01 (18.75)	91.52 (17.93)	3	6.68	0.0002	0.77
	Physical role	93.92 (20.22)	79.66 (37.39)	88.57 (27.65)	83.62 (32.77)	3	14.38	0.0000	0.94
	Bodily pain	94.84 (12.04)	88.48 (19.64)	91.07 (16.00)	90.22 (16.50)	3	9.41	0.0000	0.56
	General health	83.02 (16.33)	76.53 (16.78)	74.21 (17.96)	77.78 (17.46)	3	15.72	0.0000	0.92
	PCS	91.66 (10.78)	83.64 (17.22)	85.71 (14.16)	85.78 (14.97)	3	19.85	0.0000	0.95
	Vitality	84.29 (16.88)	75.81 (20.92)	75.92 (20.36)	77.93 (22.04)	3	13.80	0.0000	0.91
	Social functioning	89.12 (20.31)	86.51 (21.69)	85.80 (22.23)	85.05 (22.95)	3	1.95	0.1199	0.20
	Emotional role	94.77 (18.65)	85.45 (32.26)	89.28 (27.94)	84.77 (32.88)	3	8.35	0.0000	0.76
	Mental health	82.11 (16.20)	75.84 (18.75)	80.46 (18.33)	79.25 (19.14)	3	7.64	0.0000	0.53
	MCS	87.57 (12.23)	80.90 (17.31)	82.87 (16.46)	81.75 (19.46)	3	11.47	0.0000	0.69
PSQI, mean (SD). Sleep Quality	Sleep quality domain	0.54 (0.61)	0.73 (0.69)	0.67 (0.63)	0.71 (0.70)	3	5.66	0.0008	0.57
	Sleep latency domain	0.60 (0.80)	1.05 (1.10)	0.84 (0.96)	0.87 (1.02)	3	12.86	0.0000	0.89
	Sleep duration domain	0.90 (0.88)	1.09 (0.97)	1.17 (0.92)	1.18 (0.94)	3	5.89	0.0005	0.55
	Sleep efficiency domain	0.05 (0.30)	0.18 (0.54)	0.11 (0.43)	0.17 (0.56)	3	5.45	0.0010	0.72
	Sleep disturbances domain	0.71 (0.54)	0.84 (0.64)	0.80 (0.60)	0.89 (0.63)	3	4.24	0.0054	0.64
	Use of sleeping medication domain	0.05 (0.41)	0.12 (0.63)	0.10 (0.53)	0.13 (0.61)	3	1.23	0.2983	0.18
	Daytime dysfunction domain	0.37 (0.57)	0.50 (0.66)	0.45 (0.63)	0.56 (0.71)	3	4.47	0.0040	0.59
	Total score for PSQI	3.25 (2.76)	4.54 (3.80)	4.17 (3.22)	4.55 (3.78)	3	10.50	0.0000	0.86

T0: period before the pandemic. T1: mandatory preventive isolation; T2: selective isolation and responsible individual distancing; T3: selective isolation with responsible individual distancing and safe economic reactivation. SF-12v2: 12-Item Short-Form Health Survey; HRQoL: health-related quality of life PCS: physical health component summary; MCS: mental health component summary; PSQI: Pittsburgh Sleep Quality Index; ZSDS: Zung Self-Rating Depression Scale; DS: depressive symptoms; IPAQ: International Physical Activity Questionnaire; SD: standard deviation. Cohen’s *d* (d) = effect size.

**Table 3:** Differences between measurement times for sleep quality, depression and PA level (dichotomized variables).

			T0 (*n* = 354)	T1 (*n* = 354)	T2 (*n* = 280)	T3 (*n* = 174)	χ^2^	*p*	*df*	Cramer’s V
PSQI, *n* (%)	Without LSQ		262 (74.01)	186 (52.54)	163 (58.21)	95 (54.60)	39.60	0.000	3	0.201
Sleep Quality	With LSQ		92 (25.99)	168 (47.46)	117 (41.79)	79 (45.40)				
ZSDS, *n* (%).	Without DS		328 (92.66)	302 (85.31)	250 (89.29)	159 (91.38)	10.93	0.012	3	0.097
Depression	With DS		26 (7.34)	52 (14.69)	30 (10.71)	15 (8.62)				
IPAQ, n (%).	Insufficiently active	Low	78 (22.03)	240 (67.80)	140 (50.00)	66 (37.93)	155.91	0.000	3	0.366
PA level	Active		276 (77.97)	114 (32.20)	140 (50.00)	108 (62.07)				
		Moderate	128 (36.16)	76 (21.47)	86 (30.71)	51 (29.31)				
		High	148 (41.81)	38 (10.73)	54 (19.29)	57 (32.76)				
SF12v2HRQoL	Below average		350 (98.87)	339 (95.76)	275 (98.21)	163 (93.68)	14.07	0.000	3	0.11
	Above average		4 (1.13)	15 (4.24)	5 (1.79)	11 (6.32)				

T0: period before the pandemic; T1: mandatory preventive isolation; T2: selective isolation and responsible individual distancing; T3: selective isolation with responsible individual distancing and safe economic reactivation. PSQI: Pittsburgh Sleep Quality Index; LSQ: low sleep quality; DS: depressive symptoms; ZSDS: Zung Self-Rating Depression Scale; IPAQ: International Physical Activity Questionnaire. PA: physical activity. HRQoL: health-related quality of life; PCS: physical health component summary; MCS: mental health component summary.

**Table 4:** Analysis of covariance of the sleep quality and health-related quality of life adjusted for sociodemographic conditions.

	Source	*df*	*F*	*p*
Total Score PSQI Sleep Quality	Model	48	3.43	0.000
	Age	1	4.71	0.391
	Sex	1	0.00	0.954
	Socioeconomic stratum	5	3.56	0.203
	Marital status	4	1.15	0.332
	Alcohol consumption	4	4.77	0.121
	Smoker	1	13.17	0.000
	BMI	5	0.87	0.500
	Diagnosis with COVID-19	1	0.15	0.697
	Living with people at risk of COVID-19	1	0.49	0.486
SF-12v2. HRQoL	Model	29	5.06	0.000
	Age	1	2.94	0.087
	Sex	1	27.90	0.159
	Socioeconomic stratum	5	1.04	0.391
	Marital status	4	1.58	0.177
	Alcohol consumption	4	1.13	0.340
	Smoker	1	0.75	0.386
	BMI	5	4.86	0.202
	Diagnosis with COVID-19	1	1.86	0.173
	Living with people at risk of COVID-19	1	0.59	0.441

PSQI: Pittsburgh Sleep Quality Index; SF-12v2: 12-Item Short-Form Health Survey; BMI (kg/m^2^): body mass index; HRQoL: health-related quality of life.

**Table 5:** Multivariate regression post MANOVA.

	Measurement Time	Coef.	*p*	[95% Confidence Interval]
PCS	T0	91.66	0.000	90.15–93.16
	T1	−8.01	0.000	−10.13–−5.88
	T2	−5.94	0.000	−8.20–−3.68
	T3	−5.87	0.000	−8.49–−3.25
MCS	T0	87.57	0.000	85.89–89.25
	T1	−6.67	0.000	−9.04–−4.29
	T2	−4.70	0.000	−7.23–−2.18
	T3	−5.82	0.000	−8.74–−2.90
Total Score PSQI Sleep quality	T0	3.25	0.000	2.90–3.60
	T1	1.29	0.000	0.79–1.78
	T2	0.91	0.001	0.38–1.44
	T3	1.29	0.000	0.68–1.91

T0: period before the pandemic; T1: mandatory preventive isolation; T2: selective isolation and responsible individual distancing; T3: selective isolation with responsible individual distancing and safe economic reactivation; PCS: physical health component summary; MCS: mental health component summary; PSQI: Pittsburgh Sleep Quality index.

**Table 6:** Logistic regressions: associations of time with dichotomized outcomes.

	Measurement Time	Odds Ratio	*p*	[95% Confidence Interval]
IPAQ PA level	T0	0.35	0.000	2.75–4.54
	T1	0.13	0.000	0.09–0.18
	T2	0.28	0.000	0.20–0.39
	T3	0.46	0.000	0.31–0.68
PSQI Sleep Quality	T0	0.35	0.000	0.27–0.44
	T1	2.57	0.000	1.87–3.52
	T2	2.04	0.000	1.46–2.86
	T3	2.36	0.000	1.61–3.46
ZSDS Depression	T0	0.08	0.000	0.05–0.11
	T1	2.17	0.002	1.32–3.56
	T2	1.51	0.140	0.87–2.62
	T3	1.19	0.607	0.61–2.30
SF12v2 HRQoL	T0	0.01	0.000	0.004–0.03
	T1	0.87	0.117	0.78–11–7
	T2	1.59	0.492	0.42–5.98
	T3	5.90	0.003	1.85–18.82

T0: period before the pandemic; T1: mandatory preventive isolation; T2: selective isolation and responsible individual distancing; T3: selective isolation with responsible individual distancing and safe economic reactivation; IPAQ: International Physical Activity Questionnaire; PSQI: Pittsburgh Sleep Quality Index; ZSDS: Zung Self-Rating Depression Scale.

## Data Availability

The data presented in this study are only available from the corresponding author upon request. The data are not publicly available because, due to the sensitive nature of the questions asked in this study, participants were assured that raw data would remain confidential and would not be shared.

## Appendix B

**Table A1:** Cronbach’s alpha reliability coefficients of the instruments at the different measurement times.

	T0 (*n* = 354)	T1 (*n* = 354)	T2 (*n* = 280)	T3 (*n* = 174)
SF-12v2	0.77	0.79	0.78	0.82
PSQI	0.81	0.80	0.80	0.84
ZSDS	0.75	0.73	0.72	0.71

T0: period before the pandemic; T1: mandatory preventive isolation; T2: selective isolation and responsible individual distancing; T3: selective isolation with responsible individual distancing and safe economic reactivation; SF-12v2: 12-Item Short-Form Health Survey. PSQI: Pittsburgh Sleep Quality Index; ZSDS: Zung Self-Rating Depression Scale.
